# Adrenergic mechanisms of absence status epilepticus

**DOI:** 10.3389/fneur.2023.1298310

**Published:** 2023-11-22

**Authors:** Evgenia Sitnikova

**Affiliations:** Institute of Higher Nervous Activity and Neurophysiology of Russian Academy of Sciences, Moscow, Russia

**Keywords:** genetic rat model, WAG/Rij rats, spike-wave seizures, alpha2-adrenoreceptors, alpha2-wakefulness, dexmedetomidine

## Abstract

Absence status epilepticus is a prolonged, generalized absence seizure that lasts more than half an hour. The mechanisms underlying the absence of status epilepticus are still not entirely understood. In this study, the study concentrates on alpha2-adrenergic mechanisms of absence status using the WAG/Rij rat model. In this model, a prolonged spike–wave activity was associated with a specific behavioral state in transition between sedation («alpha2-wakefulness»)-resembled absence status in human patients. Pharmacological activation of alpha2-adrenoreceptors may target the locus coeruleus (presynaptic alpha2-adrenoreceptors) and the thalamic part of the seizure-generating thalamocortical system (postsynaptic alpha2B-adrenoreceptors). The duration of EEG-behavioral correlates of absence status was not dose-dependent and was predetermined by the intensity of absence seizures at baseline. This model could help scientists better understand the underlying causes of absence status and develop more effective and personalized treatments for each individual.

## 1 Introduction

Epilepsy is a neurological disorder characterized by recurrent, unprovoked seizures and paroxysmal activity in the electroencephalogram (EEG). The paroxysmal epileptic pattern in the form of spike–wave discharges is the hallmark of idiopathic generalized epilepsy (1–3). There are four main syndromes of idiopathic generalized epilepsy: childhood absence epilepsy, juvenile absence epilepsy, juvenile myoclonic epilepsy, and epilepsy with generalized tonic–clonic seizures alone (1). Absence epilepsy is a common, though mild, pediatric epilepsy syndrome that often persists into adulthood. It is characterized by sudden and brief episodes of loss of consciousness called “absence seizures” and accompanied by ~3 Hz spike–wave discharges in the EEG (1, 2, 4, 5). The incidence of childhood absence epilepsy is approximately 6.3 to 8.0 children per 100,000 per year (6).

Absence status epilepticus is a prolonged, non-convulsive seizure accompanied by more or less severe impairment of consciousness and other clinical signs, such as automatisms or subtle myoclonus, tonic, atonic, or autonomic phenomena (7–11). In contrast to convulsive status epilepticus, which must be rapidly stopped to prevent death or neurologic sequelae, non-convulsive status epilepticus (i.e., absence status) is characterized by some degree of clouding of consciousness. Absence status is often overlooked or misdiagnosed for behavioral or psychiatric disturbances. It can be evoked by toxic, metabolic, or pharmacological factors as well as by convulsive epileptic seizures (7, 10, 11, 12). The diagnostic scheme of the International League Against Epilepsy recognizes absence status as a subtype of generalized status epilepticus (13). There are two types of absence status: typical and atypical. Typical absence status occurs in patients with idiopathic generalized epilepsies. Atypical status occurs in patients with symptomatic or possibly symptomatic generalized epilepsy, such as the Lennox–Gastaut syndrome (7, 9, 10, 13). In this study, the study focuses on the typical absence status induced by alpha2-adrenergic drugs in genetic rat models of absence epilepsy and Wistar Albino Glaxo rats from Rijswijk (WAG/Rij) rats (14–16). These rats are a translational model of human absence epilepsy as they are used to study causes and treatments of absence epilepsy in humans (16–19).

The review is divided into three sections. Section 2 describes alpha2-adrenergic mechanisms and absence-related processes such as sleep and sedation. Section 3 briefly introduces rat models of absence epilepsy. Section 4 describes absence status epilepticus induced by alpha2-AR agonists in genetically prone rats.

## 2 Alpha2-adrenergic control of sleep and sedation

The noradrenaline system is known to control sleep microarchitecture, dynamics of sleep spindles, and micro-arousals during NREM sleep (20–25) The neuromodulatory profile of noradrenergic signaling during wakefulness and sleep is traditionally viewed as being unidirectional, with noradrenaline release being high during active wakefulness and it is reduced during sleep (26–28). For instance, the study of Takahashi et al. (28) demonstrated that the waking process starts with the excitation of noradrenalinergic neurons in the locus coeruleus (LC, the center of noradrenergic innervations in the brain). This leads to the excitation of waking-promoting neurons and inhibition of sleep-promoting neurons, which reinforces the waking process. The sleep process starts with a cascade of disfacilitation of waking-promoting noradrenergic neurons in LC. Hayat et al. (29) demonstrated that *“low LC activity during sleep plays a key role in mediating reduced responsiveness to sensory stimuli”* (29). More recently, optogenetic techniques and other advanced methods showed how the activity of noradrenergic LC neurons controls arousal, suggesting that sleep is an unstable process [reviewed in Osorio-Forero et al. (24)]. More specifically, (1) activity of noradrenergic LC neurons was essential for generating the 0.02 Hz fluctuations in arousal during NREM sleep; (2) noradrenergic modulation of the thalamus was necessary for generating 0.02 Hz fluctuations between spindle-rich and spindle-poor periods (23, 25).

There are three main types of ARs: alpha1, alpha2, and beta with different affinities for noradrenaline, from the highest to the lowest: alpha2 (approximately 50 nM), alpha1 (approximately 300 nM), and beta1 (approximately 800 nM) [see references in Benarroch (30), Hieble (31), Wu et al. (32)]. Agonists of alpha2-AR, such as clonidine, xylazine, medetomidine, and dexmedetomidine have strong sedative, anxiolytic, and analgesic effects and are used in clinical and veterinary practice (33–40). Alpha2-ARs couple with inhibitory G proteins (Gi/o) (41, 42) that inhibit adenylate cyclase and activate potassium channels, such as G-protein-coupled inwardly rectifying K+ channels and two-pore-domain K+ channels [see reviews (31, 43)]. The activation of potassium channels, in turn, inhibits calcium channels, leading to a decrease in neuronal excitability. The activation of alpha2A-ARs causes neuronal hyperpolarization.

Clonidine and dexmedetomidine are potent and selective agonists of alpha2-ARs that are commonly used in clinical and veterinary medicine. They exert some affinity for alpha1-AR. The selectivity ratio for alpha2-ARs vs. alpha1-ARs for dexmedetomidine is 1,600: 1 and for clonidine is 220:1. This means that dexmedetomidine has eight times higher affinity for alpha2-ARs than clonidine (39, 44, 45). This is important because the sedative effect of alpha2-AR agonists is antagonized by alpha1-AR activation. This explains why drugs with relatively low selectivity for alpha2-ARs, such as clonidine, have limited hypnotic effects (39, 46).

Alpha2-AR agonists affect the level of consciousness, causing sedation and enhancing spike–wave epilepsy along with changes in the electroencephalogram (EEG) (36, 47–49). Recently, Ballesteros et al. (50) investigated neuronal mechanisms of dexmedetomidine-induced loss of consciousness and return of consciousness in non-human primates (50). They found that the loss of consciousness induced by dexmedetomidine was abrupt similar to that during natural sleep and in epilepsy. The sedative effect of alpha2-AR agonists is related to an increase in slow-wave activity in many rat strains (Table 1), and the strongest pro-absence effect was found in genetically prone Genetic Absence Epilepsy in Rats from Strasbourg (GAERS) and WAG/Rij rats with the low doses of alpha2-AR agonists (Table 1).

**Table 1:** Effect of alpha2-AR agonists and antagonists on absence-like seizure activity in rats.

Rat strain	Drug	Rout	Dose, Volume	Effect	References
Agonists: Activation of Alpha2-ARs					
Charles River rats (body weight approximately 220 g)	Clonidine	p.o.	0.0001–0.1 mg/kg	20% of rats with spontaneous SWDs. Increase in the mean duration of SWDs. Dose 5 μg/kg: maximal effect	Kleinlogel (51)
Charles River rats (body weight approximately 220 g)	Guanfacine	p.o.	0.001–1 mg/kg	20% of rats with spontaneous SWDs. Increase in the mean duration of SWDs. Dose 1 mg/kg: maximal effect	Kleinlogel (51)
Fischer 344 rats (8–10 months old)	Clonidine	i.p.,	0.02 or 0.1 mg/kg	Increase in HVS incidence	Buzsáki et al. (52)
Fischer 344 rats (8–10 months old)	Clonidine	i.th., bilateral	0.1 or 1 nmol	Increase in HVS incidence	Buzsáki et al. (52)
Fischer 344 rats (8–10 months old)	Clonidine	i.th., unilateral	10 or 100 pmol	Increase in HVS amplitude	Buzsáki et al. (52)
Fischer 344 rats (8–10 months old)	Xylazine	i.p.,	0.5 or 2 mg/kg	Increase in HVS incidence	Buzsáki et al. (52)
Wistar (body weight 350–450 g)	Clonidine	i.p.	0.01–0.1	Increase in SWD total duration	Micheletti et al. (53)
GAERS (body weight 250–350 g, 3–4 months old)	Dex	i.c.v.	0.1, 0.5, 2.5 μg	Increase in the total SWD number, absence status epilepticus	Yavuz et al. (54)
Wistar (body weight 250–275 g)	Guanfacine	i.p.	0.004, 0.02, and 0.1 mg/kg	Increase in HVS incidence and duration. Dose 0.004 mg/kg: no effect on HVS duration	Riekkinen et al. (55)
Wistar rats (< 10 months old)	Dex	i.p.	0.005 mg/kg	Increase in HVS incidence	Yavich et al. (56)
WAG/Rij rats (body weight 320–360 g 11–12 months old).	Clonidine	i.p.	0.00625 mg/kg	Increase in SWDs incidence	Sitnikova and Luijtelaar (57)
WAG/Rij rats (6–7 months old)	Dex	i.p.	1 mg/kg	Very high dose. Decrease in total SWD number and no effect on SWD duration	Al-Gailani et al. (58)
Antagonists: Inhibition of Alpha2-ARs					
Charles River rats (body weight approximately 220 g)	Yohimbine	p.o.	0.1–10 mg/kg	Decrease in the mean duration of SWDs. Dose 1 mg/kg: maximal effect	Kleinlogel (51)
Fischer 344 rats (8–10 months old)	Yohimbine	i.p.	1 and 5 mg/kg	Decrease in HVS incidence. Dose 1 mg/kg Maximal effect	Buzsáki et al. (52)
GAERS (body weight 250–350 g, 3–4 months old)	Atipamezole	i.c.v.	1–31 μg	12 and 31 μg: decrease in SWD incidence and SWD mean duration	Yavuz et al. (59)
GAERS (body weight 250–350 g, 3–4 months old)	Atipamezole	i.c.v.	12 μg, 5 days	Decrease in total SWD duration	Yavuz et al. (59)
Wistar (body weight 350–450 g)	Yohimbine	i.p.	0.5–8 mg/kg	Reduced SWD number	Micheletti et al. (53)
Wistar (body weight 250–275 g)	Atipamezole	s.c.	0.1, 1, and 10 mg/kg	Doses 1 and 10 mg/kg: suppression of HVS Dose 0.1 mg/kg: no effect	Riekkinen et al. (55)
Wistar (body weight 280–320 g, 1.7 months old)	Atipamezole	s.c., infusion	0.1 mg/kg/h	Decrease in HVS incidence during subchronic infusion (6 days)	Jäkälä et al. (60)
Wistar rats (< 10 months old)	Atipamezole	i.p.	0.01–4 mg/kg	Decrease in HVS incidence	Yavich et al. (56)
Wistar rats (< 10 months old)	Idazoxan	i.p.	0.1–4 mg/kg	Dose <0.5 mg/kg: decrease in HVS incidence. Dose >0.5 mg/kg: no effect	Yavich et al. (56)
Wistar rats (< 10 months old)	Yohimbine	i.p.	0.1–4 mg/kg	Dose <0.5 mg/kg: decrease in HVS incidence. Dose >0.5 mg/g: no effect	Yavich et al. (56)

Dex, dexmedetomidine; HVS, high-voltage spike–wave spindles (the EEG activity similar to spike–wave discharges); SWDs, spike–wave discharges (EEG hallmark of absence epilepsy). Rout of administration: i.p., intraperitoneal; i.c., intracisternal; i.th., intrathalamic; i.c.v., intracerebroventricular; p.o., perioral; s.c., subcutaneous.

**Figure 1:**
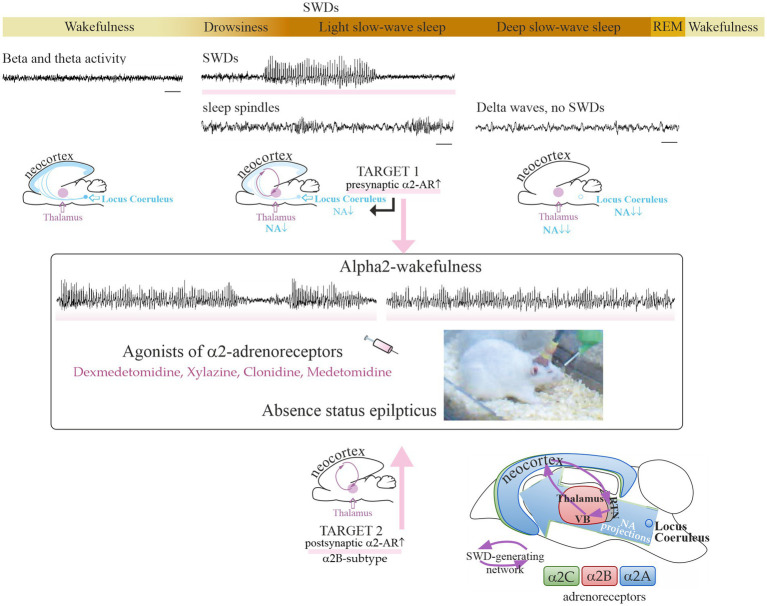
Sleep–wake continuum and noradrenalinergic mechanisms of absence epilepsy. The upper plot shows a sequential timeline of changes in vigilance, along with changes in EEG patterns. Spike–wave discharges (SWDs highlighted with rose stripes) are EEG hallmarks of absence seizures and appear spontaneously during drowsiness and light slow-wave sleep. Time bars under EEG tracks show 1 s. The SWD-generating neuronal circuit is comprised of the neocortex and the thalamus. A specific vigilance state «alpha2-wakefulness» was induced by the systemic injection of an agonist of alpha2-adrenoreceptors and accompanied by prolonged SWDs (49). The putative neuronal targets involved in the development of absence status in rat models: in the center of noradrenergic innervations, locus coeruleus (target 1), and in the thalamus (target 2). The bottom plot shows a schematic distribution of alpha2-ARs over the rat brain (65). Alpha2B-adrenoreceptors in the thalamus (target 2) might be involved in the development of absence status in epileptic rats. The reticular nucleus of the thalamus (RTN) and relay thalamic nuclei, such as the ventrobasal complex (VB), are part of the SWD-producing neuronal network. REM, rapid-eye-movement sleep. NA, noradrenaline. Partially adapted from Sitnikova et al. (65). licensed under CC BY 4.0.

Low doses of dexmedetomidine caused a pro-absence effect in rats, whereas high doses caused a reduction in SWDs and deep sedation (Table 1). In the same way, systemic application of alpha2-AR agonist clonidine in the WAG/Rij rats in a very low dose (0.00625 mg/kg) caused prolonged and recurrent spike–wave seizures in WAG/Rij rats (57). Higher doses of clonidine caused sedation [i.e., (61)]. Pharmacologically induced sedation and pro-absence effects seem to be competitive: low doses of alpha2-AR agonists in healthy rats caused light sedation and in genetically prone rats caused absence seizures up to absence status (subjects with severe epilepsy). In that regard, P. Halász introduced two important ideas: (1) *“sleep provides an excellent diagnostic tool to activate epileptic interictal and ictal manifestations.”* (62); (2) “*absence epilepsy in terms of states and functions seems to be linked to initiation of NREM sleep”* (63). Considering that rats with severe absence epilepsy showed disturbances of sleep (i.e., signs of sleep fragmentation, a higher number of micro-arousals, reduction of REM sleep [see references in Sitnikova (64))], and NREM sleep initiation mechanisms in these rats might be disturbed. As a consequence, alpha2-AR agonists in epileptic subjects might more readily elicit SWDs instead of sedation (Figure 1).

Alpha2-AR agonists, such as clonidine and dexmedetomidine have imidazole rings in their structure (66, 67), see also (42, 68). Dexmedetomidine has selectivity ratio alpha2-AR: imidazoline = 32: 1 and this ratio in clonidine = 16:1. Many regions of the brain, including the cerebral cortex, express the imidazoline subtype I2 receptor (44). Stimulation of the I2 receptors mediates a central hypotensive action, which might also account for the pro-absence effect via reducing blood pressure.

## 3 GAERS and WAG/Rij rat models of absence epilepsy

There are two well-accepted rat models of typical absence epilepsy—Wistar Albino Glaxo rats from Rijswijk, WAG/Rij (14–16, 69) and genetic absence epilepsy rats from Strasburg, GAERS (70–72). There is no answer as to which genes cause epilepsy in WAG/Rij rats and GAERS. Genetic data obtained from the WAG/Rij model for absence epilepsy show a relatively simple pattern of inheritance, with one gene determining whether an individual is epileptic or not and other genes regulating the number and duration of seizures (73). WAG/Rij rats and GAERS have been extensively used to study the molecular, cellular, and neuronal network mechanisms of typical absence epilepsy as well as to develop new drugs and anti-absence treatments [see references in Depaulis and van Luijtelaar (70), van Luijtelaar and Coenen (74), Depaulis and Charpier (75), Leo et al. (17), and van Luijtelaar and van Oijen (16)]. It was not possible to directly assess the state of consciousness in rats; therefore, the only criterion for the absence epilepsy in rats was the presence of spontaneous spike–wave discharges (SWDs) in the EEG. Earlier, we demonstrated that SWDs in WAG/Rij rats were similar to those in human patients (69); therefore, EEG-based results for absence seizures obtained in the WAG/Rij rat model have a translational value.

SWDs in WAG/Rij rats occur most frequently during the transition from wakefulness to sleep and also during light slow-wave sleep (76, 77). WAG/Rij rats have mild sleep disturbances, such as slow-wave sleep fragmentation, an increase in micro-arousals, an enhanced intermediate stage of sleep, and reduced REM sleep [see references in Sitnikova (64)]. The higher incidence of SWDs was associated with micro-arousals in WAG/Rij rat subjects with severe epilepsy at the age of 5 months (64, 78, 79). This study proved the functional links between micro-arousals and absence epilepsy, which were suggested by P. Halász in the past based on clinical data from human patients (80, 81).

The GAERS and WAG/Rij rats have face, predictive, and construct validity for typical absence epilepsy, and they are widely used in basic and preclinical studies. Predictive validity is the most important issue for preclinical studies. This means that animal models should predict the results of new human treatments, often drugs. First, it helps to ensure that the new treatment is effective and safe. Second, it allows researchers to test new treatment hypotheses (16, 82). The predictive validity of GAERS and WAG/Rij rat models was confirmed with the administration of commonly used anti-absence drugs, such as ethosuximide, levetiracetam, and valproate [for references, see (18, 82)]. The opposite, pro-absence effect of anti-epileptic drugs, such as carbamazepine and vigabatrin, was demonstrated in GAERS and WAG/Rij rat models (83, 84) and in human patients (85, 86). The WAG/Rij rat is a suitable animal model for absence epileptogenesis and anti-epileptogenesis (16, 18, 87, 88). WAG/Rij rats exhibit fully blown SWDs on their EEGs at the age of 4–5 months (symptomatic stage). The preclinical period (2–3 months of age) is characterized by significant functional changes in the neurons and neuronal networks, such as alterations in ion channels and membrane properties. This results in neuronal hyperexcitability and hypersynchronization and eventually in the generation of SWDs in the symptomatic stage (17, 88), which is common with GAERS (75).

## 4 Absence status epilepticus induced by alpha2-AR agonists in genetically prone rats

More than 30 years ago, *in vivo* studies in genetically prone rats demonstrated an increase in spike–wave activity after the application of alpha2-adrenoreceptors (alpha2-ARs), such as xylazine (52) and clonidine (53, 57, 89). Table 1 summarizes the effects of alpha2-AR agonists and antagonists on absence-like seizure activity in rats. In GAERS and WAG/Rij rats, alpha2-AR agonists caused an increase in SWDs (i.e., EEG hallmarks of absence seizures) and in other rats caused an increase in high-voltage spike–wave spindles (HVSs), which are considered absence-like seizure activity [(90) see references in Van Luijtelaar and Sitnikova (91)]. More recently, Turkish and Russian groups independently demonstrated that injections of selective alpha2-AR dexmedetomidine (Dex) caused typical absence status in the GAERS (54) and WAG/Rij rats (49, 65). Spontaneous SWDs appear during drowsiness and light slow-wave sleep (Figure 1), whereas active wakefulness (associated with beta and theta in EEG) and deep slow-wave (associated with predominant delta) are not favorable states for SWDs to occur [(76, 77, 92) see references in Kozák et al. (93) and van Luijtelaar and van Oijen (16)]. The application of alpha2-AR agonists resulted in continuous SWDs, and this pro-absence effect was the most pronounced after dexmedetomidine administration in low doses (Table 1). Antagonists of alpha2-ARs, in contrast, suppress SWDs (Table 1) putatively via an indirect effect on vigilance, i.e., behavioral excitation.

There are two primary candidate neuronal targets for alpha2-adrenergic agents inducing absence status (Figure 1): one is in the center of noradrenergic innervations in the brain, LC, and the other is in the thalamus as the part of SWD-generating thalamocortical system [some of the most well-known classical works (52, 94–96)].

Target 1 (Figure 1): the presynaptic alpha2-ARs in the noradrenergic neurons of the LC. Activation of these alpha2-ARs is known to reduce the release of noradrenaline from terminals [see refs in Poe et al. (43)]. Target neurons all over the brain receive less noradrenergic modulation via postsynaptic alpha1- and beta-ARs. This results in a reduction of vigilance and increases synchronization in the thalamocortical neuronal network that starts generating slow-wave sleep oscillations and SWDs (94, 97, 98). This is in line with L. Nelson et al.’s conclusion (2003): *“a dexmedetomidine-induced decrease in firing of noradrenergic LC neurons leads to loss of consciousness, at least in part,* via *activation of an endogenous sleep-promoting pathway”* (99).

Target 2 (Figure 1): the postsynaptic alpha2-ARs in the thalamus (52, 57). These are the subtype B receptors [see refs in Sitnikova et al. (65)]. The subtype alpha2B-AR is almost exclusively expressed in the thalamus (100–102). The thalamic alpha2B-adrenoreceptors might modulate spike–wave epilepsy, possibly through the regulation of cellular mechanisms such as the mixed Na+/K+ current (96) and AMP-activated protein kinase potentiating GABAergic signaling (103).

Alpha2-AR agonists did not induce spike–wave seizures in healthy control rats (49) probably because the receptor-binding profile of their alpha2-ARs differs from that in epileptic rats. In other words, the alpha2-AR subtypes might be differently distributed over the brains of healthy and epileptic individuals with the overexpressed thalamic alpha2B-ARs (target 2 in Figure 1). The upregulation of the thalamic alpha2B-ARs might result in absence status in genetically prone subjects, and it might be a promising molecular target for the treatment of absence epilepsy (65). Pharmacological studies are needed to confirm this hypothesis. These studies can involve both local and systemic application of drugs acting on alpha2B-ARs (as a putative molecular target) in genetically prone subjects. In order to fully understand the molecular mechanisms of absence status, it is important to consider the putative pro-absence effects of drugs with a high affinity for alpha2A and alpha2C-ARs, as well as the involvement of secondary messengers.

Our team recently investigated the alpha2-adrenergic modulation of spike–wave epilepsy in symptomatic WAG/Rij rats using systemic injections of dexmedetomidine (49, 65). We found that i.p. injections of dexmedetomidine in doses of 0.0033–0.0120 mg/kg caused a specific behavioral state, so-called «alpha2-wakefulness» (49). This state was accompanied by prolonged spike–wave seizures in the EEG (Figure 1) and might represent a behavioral state of absence status (49). The state of «alpha2-wakefulness» started 1.5–8.5 min after the injection of Dex, lasted for 1–3.9 h, and was followed by a normal sleep–wake cycle without transitioning to the deep sedation phase. During this state, rats were «trapped» in the transition state between wakefulness and sedation and showed discontinuous SWDs in EEG that were occasionally interrupted by short episodes of wakefulness or by the state with mixed EEG patterns. Interestingly, the duration of EEG-behavioral correlates of absence status was not dose-dependent and was predetermined by the intensity of absence seizures at baseline.

## 5 Limitations and future directions

In this study, the experimental model of absence status in a genetic rat model obtained using pharmacological provocation is discussed. Genton et al. (9) emphasized that absence status should be unprovoked. Provocation of the absence status in rat models is the major limitation of the proposed approach. However, it is just a model. In fact, it is important that agonists of alpha2-ARs did not induce absence seizures in non-epileptic control rats. Absence status was only found in epileptic rats with spontaneous absence seizures at baseline (49).

The pro-absence effect of low doses of dexmedetomidine was not dose-dependent, and this effect was predetermined by the intensity of absence seizures at baseline. It is assumed that the incidence of SWDs at baseline is correlated with the intensity of pharmacologically induced absence status. Future experiments are required to test the pro-absence effect of the most effective central dose of 0.008 mg/kg and two supplementary doses—0.004 and 0.012 mg/kg—in rats with different epileptic phenotypes.

It is a challenging problem for translational neuroscience in the field of epilepsy to model absence status epilepticus in rats with a translational value for human patients. The translational value of the proposed model is still in its early stages and needs more extensive ex- and pre-clinical research before it can be considered a viable option for widespread use. The starting point for modeling could be a strong pro-absence effect of agonists of alpha2-ARs.

## 6 Conclusion

The rapidly evolving field of translational neuroscience for epilepsy needs new and creative ways to model epileptic conditions. Alpha2-adrenergic mechanisms of absence status epileptics in genetically prone rats are discussed in this study. In the pharmacological model, systemic injection of the selective alpha2-adrenoreceptor agonists provoked continuous spike–wave seizures in the EEG associated with the specific behavioral state («alpha2-wakefulness») resembling absence status. The duration of absence status was predetermined by the baseline intensity of absence seizures. Alpha2-adrenoreceptor agonists induced spike–wave seizures in subclinical individuals without any prior seizures. Therefore, dexmedetomidine, which is frequently used in clinical practice, and other alpha2-adrenoreceptor agonists could help identify hidden forms of absence epilepsy that go undiagnosed and untreated. Enhancement of alpha2-adrenoreceptors via aggravation of cortico-thalamo-cortical dysfunctions can result in absence epilepsy and even absence status. It should be taken into consideration that centrally acting alpha2-adrenergic agonists have a pro-absence effect, especially in individuals with a genetic predisposition to absence epilepsy. Future studies are needed to better understand brain mechanisms of sedation, a specific behavioral state («alpha2-wakefulness») linked to absence status. In future, it will be important to examine the neurological consequences of pharmacologically induced absence status in order to better understand this complicated condition.

## Author contributions

ES: Conceptualization, Funding acquisition, Resources, Visualization, Writing – original draft, Writing – review & editing.
